# Targeting environmental determinants and settlements in public health planning for the containment of visceral leishmaniasis: a One Health perspective

**DOI:** 10.3389/fpubh.2026.1939370

**Published:** 2026-09-15

**Authors:** Saurabh RamBihariLal Shrivastava, Prateek Sudhakar Bobhate, Mohammed Kamran Shaikh, Nandkishor Bankar, Prithvi Petkar, Swapnil Inkane, Harshal Mendhe

**Affiliations:** 1 Department of Community Medicine, Datta Meghe Medical College, Datta Meghe Institute of Higher Education and Research, Nagpur, Maharashtra, India; 2 Dr. D. Y. Patil School of Allied Health Sciences, Dr. D. Y. Patil Vidyapeeth (Deemed to be University), Pimpri, Pune, Maharashtra, India; 3 Department of Community Medicine, All India Institute of Medical Sciences, Vijaypur, Jammu and Kashmir, India; 4 Department of Microbiology, Datta Meghe Medical College, Datta Meghe Institute of Higher Education and Research, Nagpur, Maharashtra, India

**Keywords:** climate change, community, education, environment, integrated vector management, kala azar, outbreaks, sandfly

## Abstract

Visceral leishmaniasis (also known as kala-azar) continues to be a major public health challenge in endemic regions. While vector control and clinical management have gained considerable attention, environmental determinants of transmission are often not prioritized in elimination strategies. In this article, we highlight gaps in surveillance that overlook environmental risks and propose their integration into routine elimination activities. At the same time, we also propose strengthening community-based monitoring, systematic environmental assessments, and harmonized data collection as tools to support existing approaches. These interventions are envisaged from a One Health perspective, underscoring the interconnected roles of human, animal, and environmental health. By integrating environmental measures with established epidemiological and clinical strategies, we propose a comprehensive approach to support the sustainability of kala-azar elimination efforts. The contribution of this perspective lies in emphasizing environmental surveillance as an essential pillar of elimination programs rather than a peripheral activity.

## 1 Introduction

Visceral leishmaniasis is a life-threatening infectious disease, with a case fatality rate of more than 95% if the condition is left untreated (1). This type of leishmaniasis is endemic in nearly 80 nations, predominantly affecting populations in South Asia and East Africa (2). The available global estimates suggest that, on average, 70,000 new cases and 25,000 associated deaths occur each year, although underreporting cannot be ruled out (2). The disease remarkably influences the socioeconomic well-being of affected populations, resulting in significant disability, reduced quality of life, and financial burdens, especially in South Asia (1). Despite advances in vector control strategies and therapeutic approaches, visceral leishmaniasis remains one of the leading neglected tropical diseases (2). While the existing literature on kala-azar predominantly focuses on early diagnosis, treatment, and vector control, there has been less emphasis on the systematic integration of environmental determinants into routine elimination planning. The current article advocates for a public health planning perspective that combines environmental surveillance, settlement-level measures, and a One Health approach to effectively contain visceral leishmaniasis.

A wide range of operational and implementation constraints can significantly affect the limited integration of environmental determinants into existing elimination activities (1, 2). To date, elimination programs have primarily focused on case detection, treatment, vector control, and outbreak response, while environmental information has been generated through separate sectors and has not been routinely linked to disease surveillance activities (2, 3). Furthermore, environmental surveillance requires sustained collection and interpretation of information regarding housing conditions, sanitation, land use, climate, and vector habitats. However, this effort can be hampered by limited resources, fragmented inter-sectoral coordination, and inadequate mechanisms for translating ecological information into public health action (1–3). These limitations cumulatively justify the need for a more integrated approach in which environmental risk assessments are incorporated into ongoing surveillance and housing-level planning rather than being treated as an additional activity.

## 2 Environmental factors and sandfly breeding

Visceral leishmaniasis is transmitted through the bite of infected female sandflies, and multiple environmental attributes influence the breeding and transmission of the infection (2). Evidence from a systematic review indicates that housing attributes (such as construction materials and structural features) are associated with the risk of leishmaniasis, although the magnitude and consistency of these associations might vary across heterogeneous settings (4). Ecological and observational studies have shown associations between environmental conditions, such as vegetation, moisture, sanitation, and the presence of suitable peridomestic habitats, and vector distribution and disease transmission (3, 5, 6). These findings support the need to implement integrated vector control measures while recognizing that their effectiveness may depend on local ecological and epidemiological characteristics (3–6). In addition, as sandflies often breed near animals, residents in endemic regions can be educated about safe livestock housing practices (7, 8). Furthermore, the accumulation of organic matter near human dwellings may encourage vector breeding. Therefore, specific measures can be implemented to improve environmental hygiene and disrupt the life cycle of the causative vector (6).

Evidence obtained from environmental and longitudinal observational studies indicates that climatic and land-use variables may influence sandfly abundance and the spatial or temporal distribution of visceral leishmaniasis (9, 10). A recent study conducted in China revealed an association between environmental changes and an increased risk of transmission (9), while a two-year prospective study from Sri Lanka assessed the relationship between climate, land-use characteristics, and temporal variations in sandfly density (10). These findings suggest that temperature, rainfall, humidity, and land-use patterns may be useful for identifying areas of changing ecological suitability. Nevertheless, these relationships are context-specific and should not be interpreted uniformly across all settings (9, 10). Once potential ecological hotspots are identified, surveillance activities can be strengthened in these areas through active case finding, enabling the detection of potential outbreaks (8).

Furthermore, systematic monitoring of environmental variables, such as rainfall, temperature, and humidity, can provide useful insights regarding sandfly population surge to public health professionals and enable them to prepare for anticipated disease outbreaks (10). In other words, planning interventions before the monsoon breeding season makes them more effective (3, 5). Changes in land use, urbanization, and deforestation have been recognized as potential environmental determinants of vector distribution and leishmaniasis risk (6, 11). Much of this evidence is obtained from ecological, observational, or modeling studies, and the direction and magnitude of these associations may vary based on local ecological circumstances (6, 11). Therefore, environmental assessments and land-use planning should be considered complementary aspects of prevention and surveillance rather than as stand-alone approaches. Public awareness of environmental risks enables communities to remove breeding sites and adopt protective behaviors (12). Finally, recognizing environmental determinants calls for multisectoral collaboration and the adoption of a One Health approach (7, 8).

## 3 Targeting environmental determinants in public health planning

Taken together, the available ecological, observational, and review evidence suggests that vector habitat characteristics are relevant to the epidemiology of visceral leishmaniasis (3, 5, 7). However, the strength and consistency of these reported associations might vary across different settings. Environmental interventions should therefore be considered as complementary components of integrated elimination strategies rather than replacements for established interventions. In fact, to achieve disease elimination goals in a timely manner, targeting environmental attributes is of paramount importance, as it can disrupt the transmission chain (13). By focusing on environmental determinants, we can indirectly target the structural conditions that disproportionately affect marginalized populations (14). Consistent with the One Health perspective outlined earlier, we propose systematically integrating environmental determinants into elimination activities (3, 5, 6). Furthermore, this approach may help reduce dependence on pharmacological interventions and potentially mitigate the risk of drug resistance, particularly in low-resource settings (5, 15).

Poorly constructed housing in densely populated or underserved communities with poor sanitation serves as an ideal ground for sandfly proliferation and augments the risk of transmission (4, 16). Incorporating disease ecology into planning ensures that housing schemes, road construction, and urbanization do not result in the creation of new vector habitats (4). Environmental surveillance enables healthcare delivery systems to implement strategies in response to ecological changes such as migration, climate change, and deforestation (8). Even from a population perspective, it is more practical and feasible to expect community members to engage in environmental sanitation and habitat modification rather than relying solely on medical initiatives (17). In summary, public health interventions become more cost-effective and efficient when they are environmentally guided and locally targeted (7, 8, 16).

## 4 Operationalizing a One Health approach for visceral leishmaniasis surveillance

The One Health approach to eliminating visceral leishmaniasis should not remain limited to merely identifying the connection between human, animal, and environmental health (8). Monitoring human health can provide valuable information on suspected and confirmed cases, including their demographics, geographical distribution, and overall trends (8, 18). Environmental surveillance can complement these data with information on housing conditions, vegetation, soil moisture, rainfall, temperature, land use, and potential vector habitats associated with each reported suspected or confirmed case (8, 18, 19). Veterinary and entomological surveillance can provide information on animal-associated environments, livestock housing, sandfly abundance, and other indices related to vector–host interactions (8, 18, 19). The integration of collected data at the district or sub-district level could facilitate the detection of environmental or ecological hotspots and support targeted vector control, environmental management, active case detection, and community-level interventions (19, 20).

To make these surveillance system operational, the preferred approach could be through developing a shared surveillance platform or dashboard, defining standard environmental and entomological indices, reporting pathways, and periodic multidisciplinary review meetings (involving public health, veterinary, environmental, sanitation, housing, and local administration) (21, 22). For instance, an increase in vector density or favorable climatic conditions in a specific area where recent human cases have been reported could prompt an entomological assessment, environmental inspection, active case search, and targeted vector control measures. Similarly, housing assessments could be linked to ongoing case-based surveillance to identify settlements that require additional preventive measures.

It is important to understand that the objective of integrated surveillance is not to create a parallel surveillance system but to strengthen it by incorporating relevant environmental and animal-sector information into existing elimination surveillance structures (21, 22). However, the overall feasibility of this integrated surveillance depend on data quality, interoperability between information systems, the availability of trained personnel, well-defined roles and responsibilities among stakeholders and institutions, and streamlined mechanisms for the timely translation of surveillance information into practical action (18, 22). Another important aspect is that One Health integration should initially be implemented in a pilot-phase manner using feasible indicators in high-risk areas, and this approach should be followed by periodic evaluation and progressive scale-up (21, 22).

## 5 Public health planning interventions

Acknowledging the role of environmental determinants in shaping the incidence of the disease, the need of the hour is to strengthen environmental parameters by targeting different areas (2, 4, 5, 8, 16, 23–37). These interventions have been organized based on the implementation level—namely, household, community, health system, and policy levels—and are presented in Table 1. Evidence from a cluster-randomized trial conducted in Bangladesh, India, and Nepal suggests that environmental modification through wall plastering can reduce indoor sandfly density, although the reported effect was smaller and less consistent than that observed with indoor residual spraying (4). In this regard, it is recommended to seal existing cracks and plaster walls to minimize resting sites for vectors, particularly at the household level (4). Programmatic experiences from India have demonstrated that environmental and settlement-level measures, such as plastering walls and sealing cracks, can be incorporated into elimination activities. However, the quality of implementation, coverage, monitoring, and integration with routine surveillance continue to remain important determinants of program effectiveness (23). In addition, specific environmental checklists, which include criteria such as wall integrity, dampness, and waste pits, have been incorporated into program monitoring in India to help sustain recent progress (24). Similarly, a straightforward environmental score based on housing materials, drainage, vegetation, and other relevant factors has been incorporated into national guidelines in Nepal to track visceral leishmaniasis. (25) Housing layouts should be designed to ensure proper spacing and airflow, preventing the humid and dark conditions that are favored by sandflies (26).

**Table 1:** Environmental interventions for visceral leishmaniasis elimination, organized by implementation level.

Implementation level	Recommended intervention	Intended programmatic contribution
Household	Plaster walls, seal cracks, improve housing materials, and ensure adequate ventilation and spacing	Potential reduction in sandfly resting sites and indoor vector exposure
	Maintain adequate separation between animal shelters and human dwellings	Potential reduction in human–vector/host contact
	Regular removal of organic waste and maintenance of household surroundings.	Reduction in potential peridomestic breeding/resting sites
Community	Routine cleaning and maintenance of drains; address waterlogging	Potential reduction in suitable vector habitats
	Village−/community-level waste collection, disposal, and composting	Improved environmental hygiene and source reduction
	Local-language behavior change communication and participatory campaigns	Increased community participation in environmental source reduction
	Community clean-up activities involving youth and school groups	Sustained environmental management and protective behaviors
Health system	Routine monitoring of housing conditions, sanitation, vegetation, climate, and vector habitats	Earlier identification of environmental risk and potential hotspots
	Monitor rainfall, temperature, and humidity; develop early-warning approaches	Improved preparedness for periods of increased ecological suitability
	Use satellite imagery and geographic information systems to identify and map environmentally vulnerable areas	More targeted surveillance and resource allocation
	Link environmental indicators with routine epidemiological surveillance	More comprehensive risk assessment
Policy/Intersectoral	Include visceral leishmaniasis environmental-risk assessment in rural development and housing plans	Prevention of environmentally vulnerable settlements
	Establish multi-sectoral task forces involving health, housing, sanitation, agriculture, and environmental sectors	Improved One Health coordination
	Incorporate housing and environmental indicators into monitoring and evaluation frameworks	More systematic monitoring of environmental risks
	Combine environmental management with indoor residual spraying and personal protection measures	Complementary vector control and elimination support

Furthermore, access to well-constructed public or individual latrines should be ensured to prevent open defecation and the accumulation of organic and moist waste (27). One of the associated strategies would be to design monitoring and evaluation frameworks that incorporate environment-specific indices, such as the housing quality index and breeding-site prevalence (28). The next set of interventions focuses on sanitation and environmental management, such as developing a village-level mechanism to ensure the collection and disposal of solid waste, thereby discouraging the breeding of sandflies (17). This effort must be supported by routine cleaning of drainage systems to prevent waterlogging and the accumulation of moist soils in regions that have ideal environmental attributes to promote sandfly proliferation (13, 17). In addition, guidelines and regulations should be introduced to house livestock at a safe distance from human dwellings to prevent vector–host proximity (8). Climate-informed early-warning approaches could be explored to identify periods or areas with favorable conditions for sandfly proliferation and to assist in preparedness. However, the predictive accuracy and the effect on outbreak prevention require further evaluation (29).

There is an immense need to develop guidelines that mandate assessments of kala-azar risk in any rural development or housing plan (30). In addition, integrated vector management, including a combination of indoor residual spraying, environmental management, and personal protection measures, must be strengthened and incorporated into routine health programs (2, 5). Environmental surveillance may be strengthened with the help of satellite images to characterize vegetation, water bodies, soil types, and land-use patterns, which can assist in the identification of areas that may have a potentially high risk of vector transmission (31). However, the predictive performance and operational utility of these approaches might vary across different settings and should be evaluated before expansion. In addition, villages and settlements can be categorized based on vector density and environmental risk to facilitate the implementation of targeted interventions (32). Collaboration among meteorological, environmental, veterinary, and health authorities can facilitate the integration of climatic, ecological, entomological, and epidemiological information for better risk forecasting and the rational allocation of resources (2, 8). Evidence base, implementation considerations, and prioritization of environmental interventions for the elimination of visceral leishmaniasis are presented in Table 2.

**Table 2:** Evidence base, implementation considerations, and prioritization of environmental interventions for visceral leishmaniasis elimination.

Intervention	Evidence base	Anticipated impact	Feasibility	Priority
Housing improvement/wall plastering	Systematic review + cluster-randomized evidence demonstrating reductions in sandfly density	Moderate-High	Moderate	High
Environmental sanitation/waste management	Observational/environmental evidence; intervention evidence remains limited	Moderate	High	High
Drainage and waterlogging management	Ecological/observational evidence	Moderate	Moderate–High	High
Community environmental clean-up	Community-based/intervention evidence is limited and heterogeneous	Moderate	High	Moderate–High
Environmental surveillance integrated with case surveillance	Programmatic and observational evidence; effectiveness depends on implementation	High potential	Moderate	High
Climate monitoring/early-warning	Ecological, longitudinal, and modeling evidence	Moderate-High potential	Moderate	Moderate
GIS/satellite environmental mapping	Geospatial/modeling evidence; limited direct evidence of transmission reduction	Moderate potential	Moderate	Moderate
Environmental checklists/indicators	Programmatic experience; limited evidence of direct transmission impact	Moderate potential	High	Moderate–High
Multisectoral one health surveillance	Strong conceptual/policy rationale; limited VL-specific effectiveness evidence	High potential	Moderate–Low	Moderate
Development/housing environmental risk assessment	Ecological/planning rationale; limited direct effectiveness evidence	High potential	Moderate–Low	Moderate

The evidence base reflects the predominant type of evidence available and is not based on formal quality assessment. Prioritization is qualitative rather than based on empirically estimated effect sizes or cost-effectiveness.

The implementation of these interventions may face substantial constraints, especially in endemic and resource-constrained settings. A lack of financial support could limit access to environmental monitoring equipment, geospatial technologies, transportation, and resources for improvements in housing and sanitation. On the other hand, a shortage of trained personnel with expertise in environmental surveillance, vector ecology, geospatial mapping, and related fields may limit local health authorities’ abilities to implement and/or sustain these activities. The fragmentation of surveillance systems can interfere with timely data sharing and coordinated decision-making. At the same time, differences in institutional priorities, reporting systems, and accountability measures may pose additional coordination challenges. This calls for the need to ensure that implementation remains phase-wise and context-specific, with priority given to high-risk areas. In addition, the use of simple and standardized environmental indicators can be preferred for incorporation into existing surveillance platforms.

To ensure these interventions are sustainable, we must invest in behavior change communication using local languages, wherein planned activities establish the association between environmental factors and disease transmission (33). In addition, Panchayati Raj institutions must be involved in monitoring local environmental determinants and activities aimed at mitigating the overall burden of disease. Moreover, the topics related to vector ecology and environmental hygiene should be integrated into the school curriculum, especially in endemic areas, to improve overall knowledge (34). We can even initiate community clean-up campaigns by involving youth and school groups and improve sanitation in these areas (35). The elimination program in Bangladesh has provided evidence on how community education has directly supported environmental actions, such as household cleaning, waste removal, and the screening of damp resting sites, thereby reinforcing case-finding and vector control strategies (36). Bhutan’s national program has transitioned toward consolidation and maintenance, with the detection of active and reactive cases, along with rapid household repairs and yard clean-up activities (37). The potential mapping of environmental determinants, proposed interventions, expected outcomes, and responsible primary stakeholders is presented in Table 3 (12–17, 23–30).

**Table 3:** Environmental determinants, interventions, and expected outcomes for sustaining kala-azar elimination.

Environmental determinants	Proposed interventions	Expected outcomes	Primary responsible stakeholders
Poor housing (mud walls, cracks, and thatched roofs)	Use plastered brick/concrete; seal wall cracks; improve ventilation and spacing	Fewer sandfly resting sites; reduced indoor vector density	Housing and Urban Development; Municipalities; Community; Health Sector
Inadequate drainage and stagnant water	Construct/maintain drainage systems; regular cleaning of waterlogging areas	Less vector breeding habitat; lower risk of new transmission foci	Municipalities; Public Works; Local Governance (Panchayati Raj)
Accumulation of organic waste near households	Community-based waste disposal and composting; routine municipal collection	Reduced peridomestic breeding sites; cleaner surroundings	Sanitation/Waste Management Dept.; Community Organizations; Municipalities
Deforestation and unplanned urbanization	Environmental impact assessments; settlement planning with green buffers	Prevention of new vector habitats; safer urban growth	Environmental Agencies; Urban Planning Authorities; Local Governance
Peridomestic vegetation and animal shelters close to houses	Strategic placement of animal shelters; vegetation management around homes	Lower human–vector contact; safer living environment	Animal Husbandry/Veterinary Sector; Community; Health Sector
Lack of community awareness	Health education; participatory campaigns; school-based programs	Community engagement in source reduction; sustained protective behaviors	Health Sector (IEC/BCC); Education Department; Community Leaders/NGOs
Weak cross-sector collaboration	Multi-sectoral task forces (health, housing, sanitation, and agriculture); One Health platforms	Integrated planning; more resilient elimination programs	Ministry of Health; Ministry of Environment and Forests; Agriculture; Local Governance

## 6 Conclusion

In conclusion, a wide range of environmental attributes cumulatively determine the incidence of visceral leishmaniasis. This calls for the need to adopt a comprehensive approach, wherein different environmental parameters are targeted to reduce the incidence of the disease in settlements and vulnerable populations. However, the implementation of environmental surveillance and related interventions should be phased and context-specific, particularly in low-resource settings. Future research should focus on validating geospatial and microclimatic predictive models and evaluating the cost-effectiveness of structural modifications compared to indoor residual spraying. Operationalization of One Health governance can bridge implementation gaps between public health, sanitation, and urban planning.
